# Mutational analysis of SARS-CoV-2 variants of concern reveals key tradeoffs between receptor affinity and antibody escape

**DOI:** 10.1371/journal.pcbi.1010160

**Published:** 2022-05-31

**Authors:** Emily K. Makowski, John S. Schardt, Matthew D. Smith, Peter M. Tessier

**Affiliations:** 1 Pharmaceutical Sciences, University of Michigan, Ann Arbor, Michigan, United States of America; 2 Biointerfaces Institute, University of Michigan, Ann Arbor, Michigan, United States of America; 3 Chemical Engineering, University of Michigan, Ann Arbor, Michigan, United States of America; 4 Biomedical Engineering, University of Michigan, Ann Arbor, Michigan, United States of America; University of Technology Sydney, AUSTRALIA

## Abstract

SARS-CoV-2 variants with enhanced transmissibility represent a serious threat to global health. Here we report machine learning models that can predict the impact of receptor-binding domain (RBD) mutations on receptor (ACE2) affinity, which is linked to infectivity, and escape from human serum antibodies, which is linked to viral neutralization. Importantly, the models predict many of the known impacts of RBD mutations in current and former Variants of Concern on receptor affinity and antibody escape as well as novel sets of mutations that strongly modulate both properties. Moreover, these models reveal key opposing impacts of RBD mutations on transmissibility, as many sets of RBD mutations predicted to increase antibody escape are also predicted to reduce receptor affinity and vice versa. These models, when used in concert, capture the complex impacts of SARS-CoV-2 mutations on properties linked to transmissibility and are expected to improve the development of next-generation vaccines and biotherapeutics.

## Author summary

Machine learning is a powerful predictive tool that is well suited for diverse infectious disease applications. In this study, we apply machine learning to comprehensively predict the impact of mutations in the SARS-CoV-2 receptor-binding domain on both receptor affinity, which mediates viral infectivity, and escape from human serum antibodies, which mediates virus neutralization. These methods identify key mutations in current and former SARS-CoV-2 Variants of Concern, and predict novel high-risk variants that may warrant further consideration for vaccine and therapeutic development. Moreover, these models provide a valuable framework for future investigations aimed at understanding and mitigating COVID-19, especially as continued viral evolution remains a key global health threat.

## Introduction

The coronavirus pandemic has devastated mankind since 2019, and it is unclear when it will end given that the virus is expected to become endemic. Rapid development, approval, and distribution of vaccines has provided significant protection to vaccinated individuals. However, as immunity from both vaccination and natural infection wanes, new widespread variants with increased transmissibility threaten additional waves of devastation [1]. In particular, mutations in the receptor-binding domain (RBD) of the spike (S1) protein have demonstrated increased transmissibility through multiple mechanisms, including by i) increasing the affinity of the RBD for its cognate receptor, angiotensin-converting enzyme 2 (ACE2) [2–5] and ii) reducing RBD binding to human serum antibodies elicited by natural infection or vaccination [6–9]. For example, increased ACE2 affinity due to RBD mutations has been linked to increased transmissibility for viral lineages carrying the spike protein mutation D614G found in all current and former Center for Disease Control and Prevention (CDC) ‘Variants of Concern’ [10]. Herein, we refer to all current and former CDC Variants of Concern simply as VOCs. Likewise, reduced human serum antibody binding due to RBD mutations is linked to increased transmission for variants with the K417N/T, E484K, and N501Y mutations found in the Beta and Gamma variants, which have been shown to have increased breakthrough infection rates in vaccinated individuals [11, 12]. Either of these two mechanisms, or a combination thereof, may result in increased infection rates in unvaccinated or even vaccinated individuals, which has the potential to facilitate additional viral evolution and further increases in transmissibility. Therefore, it is of great interest to accurately predict novel RBD mutations (and combinations thereof) that confer increased transmissibility. Such predictions may be useful to inform vaccine and biotherapeutic development and guide global health decisions.

Experimentally, multiple studies have reported impressive progress characterizing the impact of RBD mutations on ACE2 and human serum antibody affinity [2, 6]. These approaches generate RBD libraries in which one or more RBD sites are mutated to the other 19 amino acids. Each RBD mutant protein in the library typically has multiple mutated sites (2–10 mutations per RBD protein). The resulting libraries (~100,000 RBD variants) are displayed on the surface of yeast, facilitating high-throughput screening via quantitative cell sorting. This approach enables library sorting against different concentrations of ACE2 to evaluate the impact of RBD mutations on ACE2 affinity and different dilutions of human plasma samples from convalescent patients to evaluate the impact of RBD mutations on human serum antibody binding [2, 6]. While the resulting datasets are impressive in their size, they are small in comparison to the maximal mutational diversity for all possible sets of mutations in the RBD (20^201^ or 10^261^ variants), containing only ~0.3% of possible RBD variants with two mutations.

**Fig 1:**
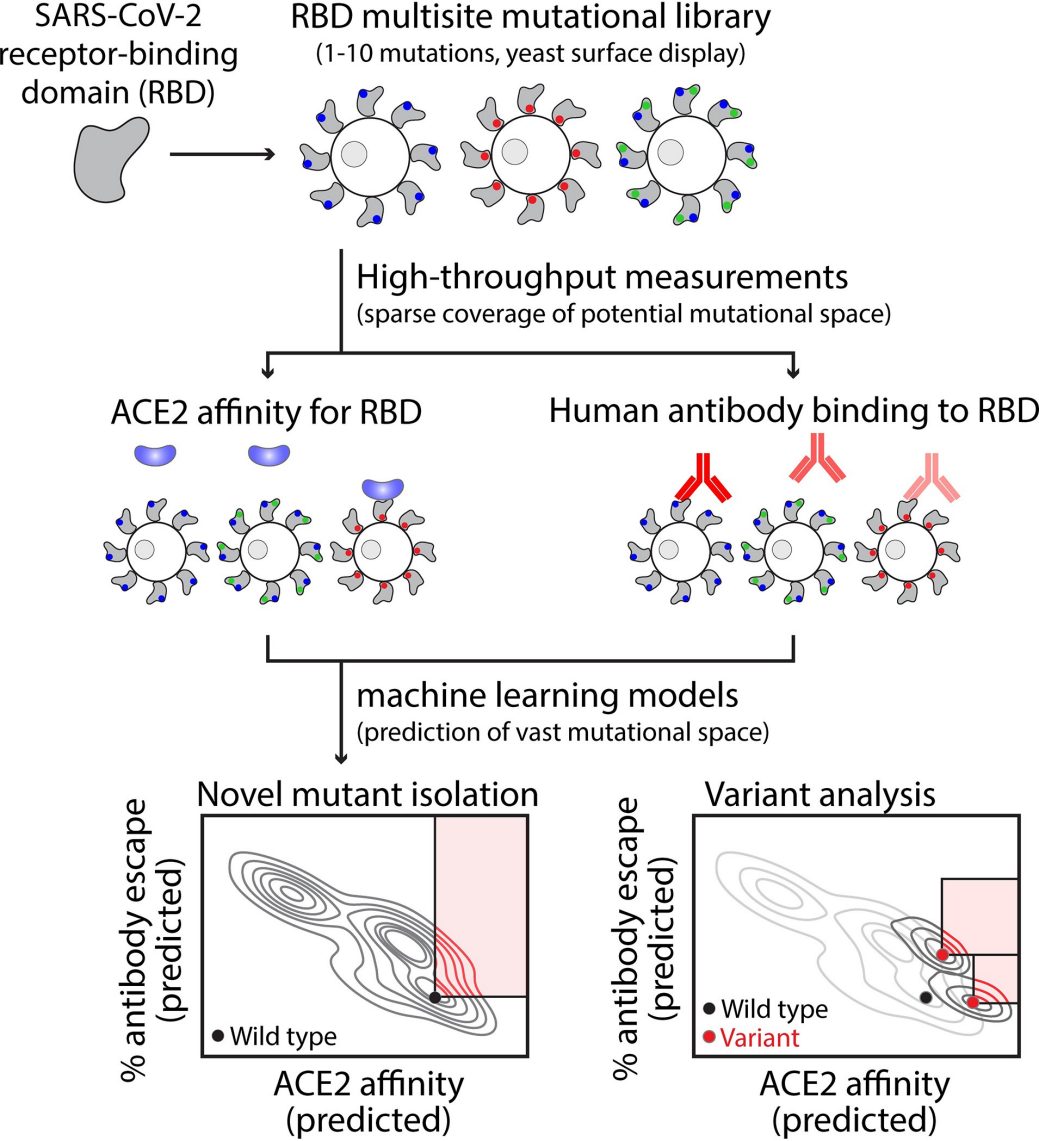
Overview of the development of machine learning models for predicting mutations in the receptor-binding domain of SARS-CoV-2 that increase transmissibility. Machine-learning models were trained and tested on large but sparsely sampled experimental datasets that characterize the impact of single and multisite RBD mutations on ACE2 affinity and human serum antibody binding (>100,000 RBD variants with 1–10 mutations). The relative binding levels of human serum antibodies to RBD mutants were converted into % human serum antibody escape values as 100% minus the % antibody binding to mutant RBD relative to wild-type RBD. It is important to consider the impacts of RBD mutations on both properties because ACE2 affinity strongly impacts viral infectivity and human antibody binding strongly impacts viral neutralization. The two models were employed to predict mutations, and combinations thereof, that increase ACE2 affinity, human serum antibody escape or both for the vast mutational space that is much larger than what is possible to evaluate using experimental methods. Mutations were identified that enhance transmissibility for wild-type SARS-CoV-2 as well as additional mutations that further enhance transmissibility of several current and former CDC Variants of Concern.

Therefore, there is a critical need for computational methods that are capable of learning from extensive but sparsely sampled RBD mutant datasets and using this information to predict the impacts of RBD mutations on key properties linked to transmissibility. In this work, we generate machine learning models capable of predicting the impacts of RBD mutations on ACE2 affinity (model #1) and human serum antibody binding (model #2; Fig 1). Used in concert, the two models identify most of the individual mutations and sets of mutations in VOCs that increase transmissibility. Moreover, we report predictions of several single nucleotide RBD mutations with increased transmissibility, including additional mutations in VOCs which have not been observed to date and represent mutations of concern that may emerge in the future.

## Results

### Tradeoffs between ACE2 affinity and human serum antibody binding

Towards our goal of developing models for predicting the impact of RBD mutations on several key properties linked to transmissibility, we first evaluated two large mutational datasets [2,6]. The first set includes the ACE2 affinities for 64,617 single and multisite RBD mutants, which we used to predict the affinities of mutated RBD sequences for ACE2, and the second set includes the percentage increases in human serum antibody escape for 102,723 single and multisite RBD mutants, which we used to predict the relative binding of human polyclonal antibodies to mutated RBD sequences. The former property (ACE2 affinity) is reported as apparent association constant (*K*_*A*, *app*_) values because the experimental data were measured using bivalent ACE2 (ACE2-Fc) and the apparent affinities are much higher than those for monovalent ACE2 [2]. The latter property (% antibody escape) is simply 100% minus the percentage of binding of human serum antibodies to a given RBD mutant relative to the wild-type RBD.

**Fig 2:**
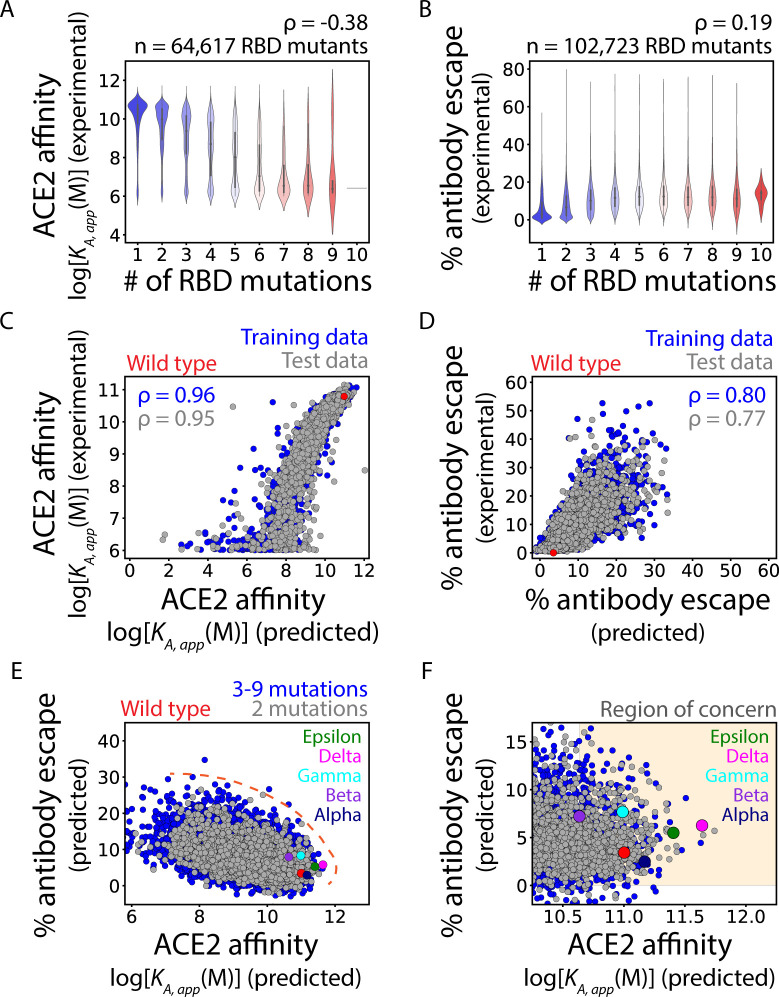
Machine learning models predict the impact of RBD mutations on ACE2 affinity and % human serum antibody escape, including for current and former CDC Variants of Concern. (A, B) Increasing numbers of RBD mutations generally (A) reduce ACE2 affinity and (B) increase % antibody escape. (C, D) Machine learning models predict the impact of RBD mutations on (C) ACE2 affinity and (D) % antibody escape for the training and test datasets with 1–10 RBD mutations. (E, F) The two models reveal natural tradeoffs between the impact of RBD mutations on ACE2 affinity and % antibody escape. The predictions for wild-type SARS-CoV-2 are highlighted in red, while the predicted values for VOCs are highlighted in different colors (see legend). In (A), (C) and (E-F), the ACE2 affinities are reported as log[*K*_*A*,*app*_ (M)] values and higher values reflect higher affinity. The affinities are apparent values because the experimental datasets were obtained using bivalent ACE2 (ACE2-Fc), which results in much higher apparent affinity than that observed for monovalent ACE2. In (A-D), Spearman’s ρ values are given. In (E), the Pareto frontier that corresponds to the maximal level of antibody escape at each ACE2 affinity is indicated by the hashed red line. In (F), the ‘Region of Concern’ is defined as ACE2 affinity predictions greater than that for the Beta variant and % human antibody escape predictions values >0%. In (C-D), model performance metrics are averages of tenfold cross-validation. In (C-F), each plot shows a randomly selected subset of 5,000 RDB mutants.

We first evaluated the impact of the number of RBD mutations on ACE2 affinity and human serum antibody binding (Fig 2). We found that increasing numbers of RBD mutations were correlated with reduced ACE2 affinity (Spearman’s ρ of -0.38), which is logical assuming there is progressive disruption of the functional RBD epitope as the number of mutations is increased (Fig 2A). Conversely, increasing numbers of RBD mutations were correlated with increased antibody escape (Spearman’s ρ of 0.19) (Fig 2B). Together, these findings demonstrate a strong tradeoff between ACE2 affinity and human serum antibody escape, which emphasizes the importance of considering both properties when predicting highly transmissible variants.

### Machine learning models predict the impacts of multisite RBD mutations

We next sought to develop models for predicting the impacts of single and multisite RBD mutants on ACE2 affinity and human serum antibody escape (Fig 2C–2F). We performed extensive preliminary analysis of various algorithms and feature sets, as summarized in S1 Table. For the ACE2 affinity dataset, we found that a simple binary encoding of the RBD sequences as one-dimensional vectors (one-hot encoding), and incorporation of these features into a Ridge Regression model, led to the prediction of ACE2 affinities that were the most strongly correlated with the experimental measurements. The Spearman’s ρ and mean absolute percent error (MAPE) values for the correlations between the predictions and experimental measurements were favorable for both the training set (ρ of 0.96 and MAPE of 3.7%) and test set (ρ of 0.95 and MAPE of 5.2%; Fig 2C). Moreover, the model predictions of ACE2 affinities were strongly correlated with conventional affinity measurements for a set of nine single RBD mutations that were experimentally characterized on an individual basis (Spearman’s ρ of 0.87; S1 Fig) [2].

We also observed strong, but modestly reduced, correlations for models developed to predict the impacts of RBD mutations on human serum antibody escape [ρ of 0.80 and mean absolute error of 3.0% (training set) and ρ of 0.77 and mean absolute error of 3.2% (test set); Fig 2D]. The modestly reduced performance of the human serum antibody model relative to the ACE2 model may reflect the more complex and heterogenous nature of the human serum antibody data obtained using samples from several patients at multiple time points.

We also repeated model training and testing using individual replicate measurements for ACE2 affinity and individual human serum samples for antibody escape; S2 Fig to compare them to the combined datasets we used in our initial analysis (Fig 2). For ACE2 affinity, we observed strong model performance for both replicates (Spearman’s ρ of 0.96 for both replicates). We observed more variable performance for the antibody escape models trained on data from each of the eleven human serum samples (each of which are an average of two or three time points after viral infection; Spearman’s ρ of 0.34–0.62).

Another potential concern of our models is that they may not be able to accurately capture the impacts of multiple RBD mutations that are not observed together in the training sets. To test this issue, we repeated the model training but modified the training and test sets. For the training sets, we removed all double RBD mutants as well as all triple and higher order mutants that included the observed double mutants. After the models were trained, they were tested only on the double RBD mutants, which were never observed together in the training sets. Encouragingly, we found that the accuracies of the models were similar as before, as the correlations for the double mutant predictions were similar to those for the original models (S3 Fig). For example, the correlations for the double RBD mutants (Spearman’s ρ of 0.93 for ACE2 affinity and 0.68 for antibody escape) were similar to those for the original test sets with diverse sets of single, double, and higher order RBD mutations (ρ of 0.95 for ACE2 affinity and 0.79 for antibody escape). We repeated this same analysis for triple and quadruple RBD mutations and found similar results (S3 Fig). Collectively, these findings suggest that the models can accurately predict the impact of combinations of RBD mutations on ACE2 affinity and antibody escape, even in cases where the combinations of RBD mutations were not observed in the training sets.

### RBD mutants with increased predicted risk for high transmissibility

We next performed multi-objective (Pareto) optimization to predict RBD mutations that would either increase ACE2 affinity or human antibody escape or both (Fig 2E). As expected, the impact of RBD mutations on ACE2 affinity and human serum antibody escape exhibited an inherent tradeoff, as illustrated by a strong negative correlation between the two properties (Spearman’s ρ of -0.45). This relationship was observed both for double RBD mutants and RBD mutants with 3–9 mutations. Encouragingly from a health perspective, a vast majority of RBD mutations and combinations thereof are unlikely to confer large gains in either property without causing reductions in the other property because of the conflicting impacts of RBD mutations on both properties.

However, there is a subset of mutations, including those in VOCs, that display increases in one or both properties relative to wild type (Fig 2E). The Pareto frontier (Fig 2E, orange dashed line), which describes the maximum ACE2 affinity that is possible at each level of human serum antibody escape, enables the identification of RBD mutants with increased risk for enhanced viral transmissibility. The region surrounding the Pareto frontier is highly populated by double RBD mutants, confirming that these sequences are of great interest due to their high risk of increased transmissibility. Notably, RBD mutants corresponding to several VOCs are also located near the Pareto frontier. The Alpha, Epsilon, and Delta variants are predicted to display increased ACE2 affinity (*K*_*A*, *app*_ values of 1.5x10^11^ to 4.4x10^11^ M respectively relative to 1.0x10^11^ M for wild type) while maintaining similar levels of escape from human serum antibodies (2.5–6.3% relative to 3.4% for wild type). In contrast, the Beta and Gamma variants are predicted to display increased escape from human antibodies (7.2–7.7% relative to 3.4% for wild type) while maintaining similar ACE2 affinities (*K*_*A*, *app*_ values of 4.3x10^10^ to 9.8x10^10^ M relative to 1.0x10^11^ M for wild-type). The predictions for VOCs are summarized in Table 1.

**Table 1:** Machine learning predictions of the impact of RBD mutations in current and former CDC Variants of Concern on ACE2 affinity and human serum antibody escape.

Variant	RBD Mutations	Predicted ACE2 affinity (*K*_*A app*_, M)	Predicted antibody escape (%)
Wild type		1.00 x 10^11^	3.4
Alpha	N501Y	**1.48 x 10** ^**11**^	2.5
Beta	K417N, E484K, N501Y	4.32 x 10^10^	**7.2**
Epsilon	L452R	**2.54 x 10** ^**11**^	**5.5**
Gamma	K417T, E484K, N501Y	9.76 x 10^10^	**7.7**
Delta	L452R, T478K	**4.37 x 10** ^**11**^	**6.3**

Predictions of increased ACE2 affinity or human serum antibody escape or both relative to wild-type SARS-CoV-2 are indicated by bold values. ACE2 affinities are reported as apparent values because the experimental datasets were obtained using bivalent ACE2 (ACE2-Fc), which results in much higher apparent affinities than those observed for monovalent ACE2.

We also performed a separate analysis for the Omicron (B.1.1.529) variant, which has recently emerged as the predominant viral strain. Due to the large number of RBD mutations (15) relative to the number of mutations in our training sets (1–10 mutations), it was not possible to use our models to accurately predict the properties of the Omicron variant. However, we evaluated the individual impacts of the 15 RBD mutations on ACE2 affinity and antibody escape (Table 2). Interestingly, this analysis predicted five RBD mutations (G339D, N440K, S477N, T478K and N501Y) that enhance ACE2 affinity, and all five mutations have been independently confirmed to increase ACE2 affinity [13, 14]. Moreover, most of the RBD mutations are also predicted to increase antibody escape, particularly the E484A mutation that has been shown to substantially decrease the neutralization activity of human convalescent sera [8].

**Table 2:** Predicted impacts of individual RBD mutations in the Omicron (B.1.1.529) variant on ACE2 affinity and human antibody escape. Predictions of increased ACE2 affinity or human antibody escape are indicated by bold values. Named variants in bold are current and former Variants of Concern.

Mutation	Predicted ACE2 affinity (*K*_*A app*_, M)	Predicted antibody escape (%)	Observed in other named variants
Wild type	1.00 x 10^11^	3.4	
G339D	**1.44 x 10** ^**11**^	**3.5**	
S371L	5.69 x 10^10^	**3.9**	
S373P	8.35 x 10^10^	**5.5**	
S375F	1.51 x 10^10^	**5.2**	
K417N	2.74 x 10^10^	**4.3**	
N440K	**1.45 x 10** ^**11**^	2.9	B.1.36
G446S	2.64 x 10^10^	**5.3**	
S477N	**1.13 x 10** ^**11**^	2.8	
T478K	**1.73 x 10** ^**11**^	**4.2**	**B.1.617.2**
E484A	5.18 x 10^10^	**11.2**	
Q493R	6.04 x 10^10^	**4.1**	
G496S	1.67 x 10^10^	**5.0**	
Q498R	9.00 x 10^10^	**3.7**	
N501Y	**1.48 x 10** ^**11**^	2.5	**B.1.1.7, B.1.351, P.1**
Y505H	1.05 x 10^10^	**4.8**	

### Prediction of concerning double RBD mutations in wild-type SARS-CoV-2

We next sought to analyze the RBD double mutants that increased ACE2 affinity or human serum antibody escape or both relative to wild-type RBD and the RBDs of VOCs (Fig 2F). This region of concern was defined by ACE2 affinities (K_*A*, *app*_ values) higher than that of Beta (4.3x10^10^ M), which is modestly lower than wild type (1.0x10^11^ M), as the lower bound of concerning ACE2 affinities. We also chose a lower bound of 0% predicted human serum antibody escape. Together, these constraints on ACE2 affinity and antibody escape predictions constituted the ‘region of concern’ (Fig 2F).

**Fig 3:**
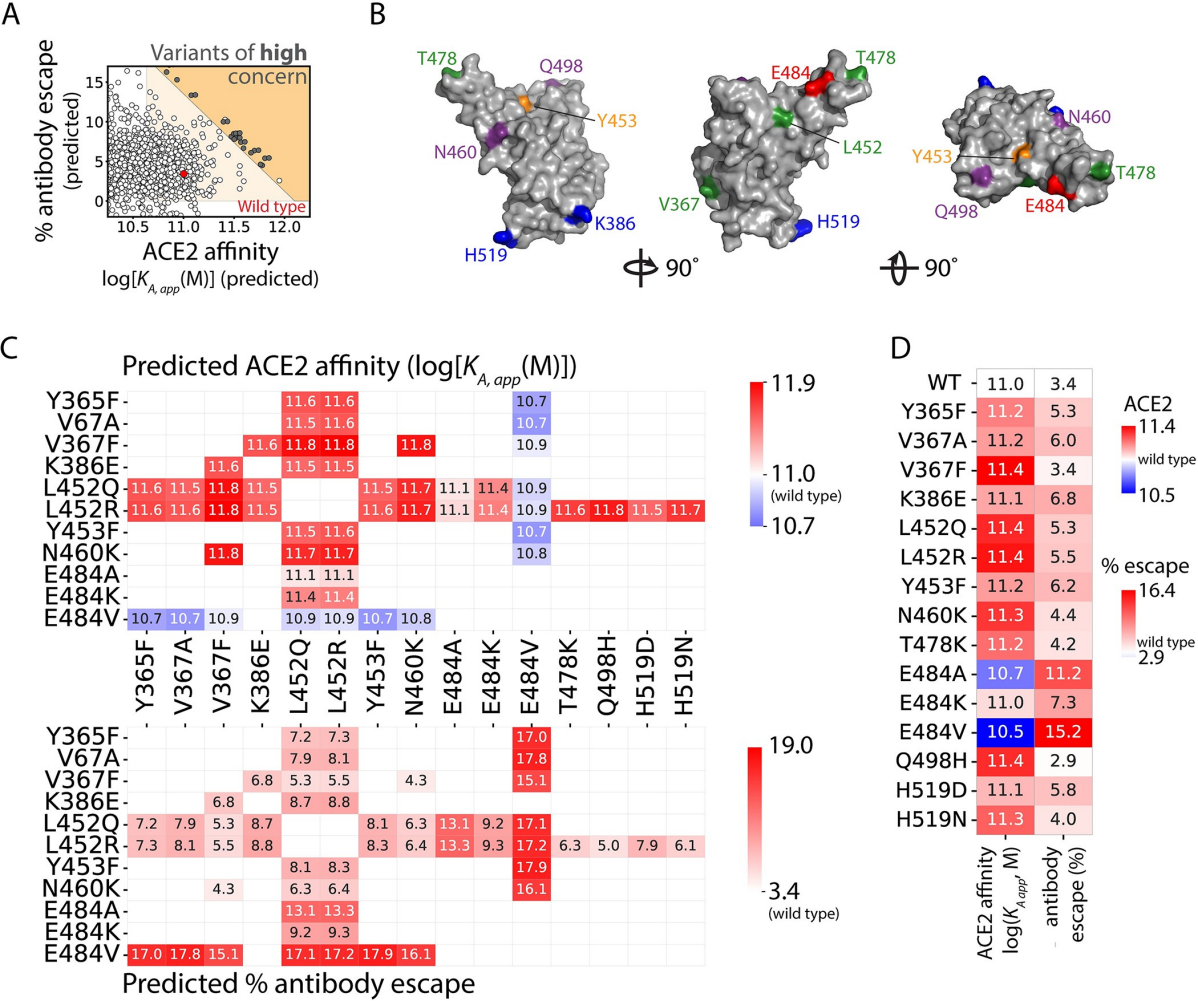
Identification of SARS-CoV-2 RBD double mutants at the Pareto frontier with co-maximal levels of ACE2 affinity and antibody escape. (A) Twenty-nine RBD double mutants (single nucleotide exchanges, dark gray), located at the Pareto frontier, were isolated with the largest increases in ACE2 affinity or antibody escape relative to wild type (red point). The white points at the Pareto frontier required multiple nucleotide exchanges and were not considered further, and only a subset of the evaluated RBD variants (including those at the Pareto frontier) are shown for clarity. (B) Structural locations of eleven of the sixteen RBD sites mutated in the 29 RBD double mutants that resulted in the largest increases in either ACE2 affinity or antibody escape or both. (C) Structural locations of the other RBD sites that were also mutated in the 29 RBD double mutants located at the Pareto frontier. (D) Predicted values of (top) ACE2 affinity and (bottom) antibody escape for the 29 RBD double mutants located at the Pareto frontier. (E) Predicted values for the single RBD mutations observed in the RBD double mutants located at the Pareto frontier. ACE2 affinities are reported as the log[*K*_*A*,*app*_(M)]. In (B) and (C), the wild-type residues are colored red (negative charge), blue (positive charge), green (hydrophobic), orange (tyrosine) and purple (polar).

We next performed a virtual scan of all possible RBD double mutants to identify those that fall within the region of concern (S4 Fig). Approximately 5x10^5^ sequences within this region of concern were isolated and are reported in S1 Dataset. We further filtered the RBD double mutants to isolate the most concerning variants near the edge of the Pareto frontier. While we considered all possible mutations, we prioritized those that are single nucleotide exchanges given the relevance to viral evolution and the fact that all but one mutation in current and former VOCs are due to single nucleotide exchanges [15]. Only double mutants in which both amino acid substitutions could be made with single DNA nucleotide exchanges were considered in this analysis. We also removed mutations that would alter N-linked glycosylation sites due to concerns related to reduced RBD stability. Twenty-nine double mutants with the highest predicted increases in ACE2 affinity and antibody escape, located as far from wild-type behavior as possible, were isolated and named ‘variants of high concern’ (Fig 3A and S2 Dataset).

From the 29 isolated variants of high concern, we identified mutations at nine sites, all of which were solvent accessible [16]. Eleven mutations at these nine sites were present in more than one of the 29 variants; the sites of these mutations are highlighted on the RBD structure (Fig 3B). Interestingly, these sites are distributed throughout the RBD, with several localized at the ACE2-binding site. Noticeably, most (11 of 14) of the mutations identified in these double mutants have already either been reported in currently circulating variants, identified via *in vitro* studies or both (V367A, V367F, L452Q, L452R, Y453F, N460K, T478K, E484K, E484A, Q498H, and H519N) [2,6, 17–22]. These findings suggest that the predicted increases in ACE2 affinity and/or human serum antibody escape are linked to increased viral transmissibility. Of these concerning mutations, several mutations isolated in this group (L452Q, L452R, and E484K) are particularly worrisome as they are present in various named variants, including VOCs, as summarized in Table 3.

**Table 3:** Machine learning predictions of the impact of single RBD mutations on ACE2 affinity and human serum antibody escape.

Mutation	Predicted ACE2 affinity (*K*_*A app*_, M)	Predicted antibody escape (%)	Observed in other named variants
Wild type	1.00 x 10^11^	3.4	
V367F	**2.77 x 10** ^**11**^	3.4	A.23.1
L452Q	**2.32 x 10** ^**11**^	**5.3**	C.37, **B.1.1.529**
L452R	**2.54 x 10** ^**11**^	**5.5**	**B.1.617.2**, B.1.526.1, **B.1.429**, B.1.427, B.1.617.2, B.1, B.1.617.1, C.36, A.2.5
E484K	**1.08 x 10** ^**11**^	**7.3**	**P.1**, B.1.526, **B.1.351**, B.1.1.318, B.1.525, R.1, B.1.526.2, B.1.1, B.1.621, B.1, **B.1.1.7**

RBD mutations common in named SARS-CoV-2 variants, including current and former Variants of Concern (bolded), are predicted to display increased ACE2 affinity and, in some cases, increased antibody escape (as indicated by bold values).

### Predicted RBD mutations that further increase transmissibility of VOCs

**Fig 4:**
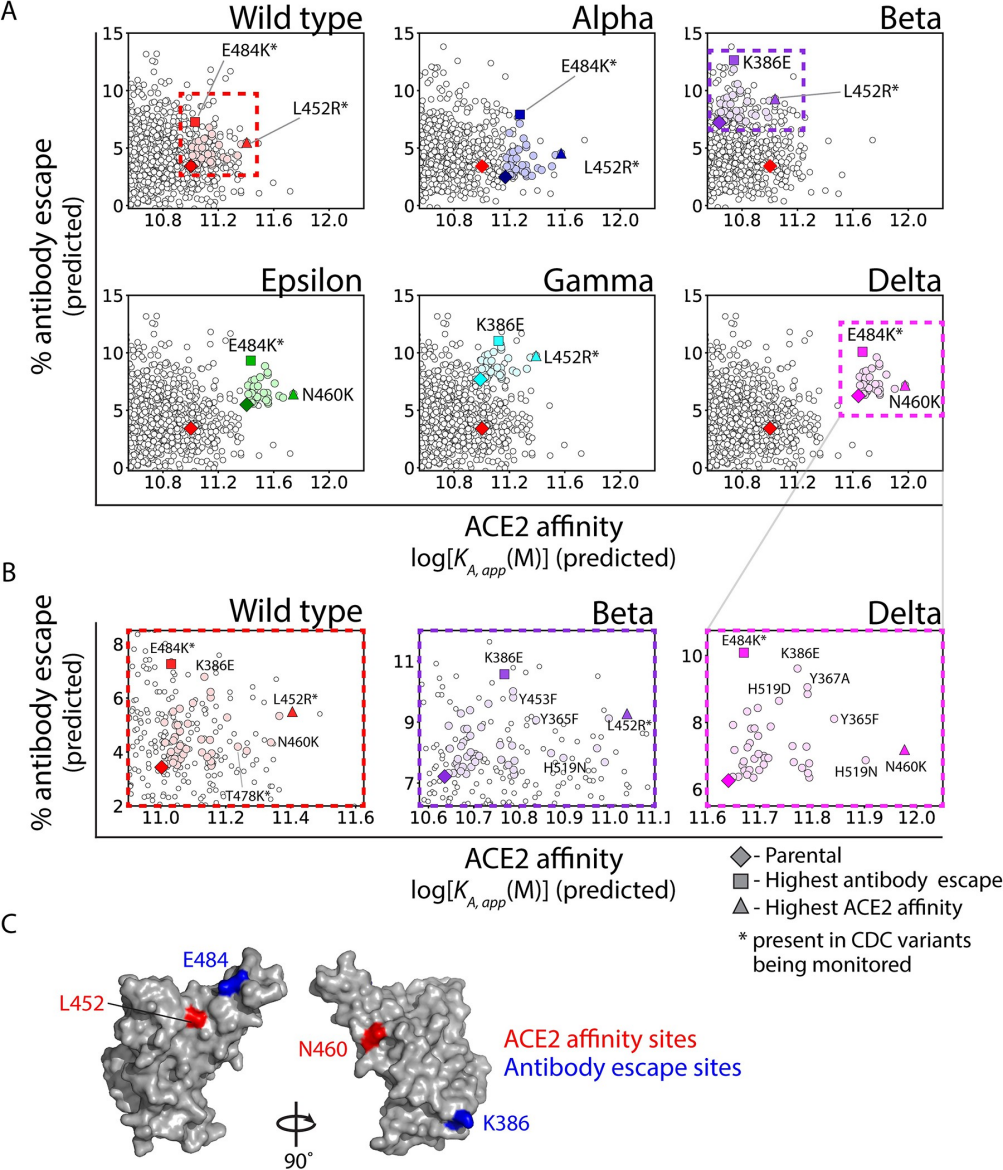
Predictions of single mutations in wild-type SARS-CoV-2 and additional single mutations in current and former CDC Variants of Concern at the Pareto frontier with the largest increases in ACE2 affinity or antibody escape. (A) Single RBD mutations that are predicted to increase ACE2 affinity or antibody escape without reducing the other property for wild-type SARS-CoV-2 and Variants of Concern. (B) Expanded view of the graphs in (A) with highlighted single RBD mutations that increase ACE2 affinity or antibody escape. (C) Location of key RBD sites that are commonly mutated to increase ACE2 affinity (L452 and N460) and antibody escape (K386 and E484). In (A) and (B), colored symbols generally correspond to single nucleotide exchange mutations (except for wild-type SARS-CoV-2 or the parental Variants of Concern), while white symbols correspond to multiple nucleotide exchanges.

As the pandemic persists and circulating variants continue to spread, there is heightened risk of additional mutations to VOCs that will further increase transmissibility. Therefore, we identified additional mutations in five VOCs (as well as wild-type SARS-CoV-2) that present the largest predicted risk to either increasing ACE2 affinity or human serum antibody escape without substantially sacrificing the other property. A virtual scan of single RBD mutations was performed for each of five key variants (Alpha, Beta, Epsilon, Gamma, and Delta) and wild-type SARS-CoV-2. In particular, we analyzed RBD mutations that could be achieved with single nucleotide exchanges without disrupting glycosylation sites and increased either ACE2 affinity or antibody escape while maintaining or increasing the other property (Fig 4A). Four mutations were found to increase transmissibility of VOCs most strongly, namely L452R and N460K for primarily increasing ACE2 affinity and E484K and K386E for primarily increasing antibody escape. Notably, L452R and E484K are present in several of the VOCs with increased transmissibility [10] Interestingly, L452K and E484K mutations are in the receptor-binding motif, while K386E and N460K are peripheral to the receptor-binding motif (Fig 4B). A summary of these mutations can be found in S3 Dataset.

Finally, we also performed a similar analysis to identify single RBD mutations that led to the largest increases in either ACE2 affinity or antibody escape for the VOCs, but no longer required that the other property be maintained or increased (S4 Fig). This analysis revealed that the RBD mutation with the largest increase in ACE2 affinity for all of the VOCs was V367F, which has been isolated and analyzed previously for its impact on infectivity and antibody escape [21]. Interestingly, this RBD mutation was confirmed to increase *in vitro* infectivity, consistent with our prediction of increased ACE2 affinity, and to reduce human serum antibody escape, which is also consistent with our predictions. Our analysis also identified a second RBD mutation, namely F456A, that was predicted to mediate the largest increase in antibody escape. This mutation has not been detected in any VOCs, which may be due to the large, predicted reduction of ACE2 affinity. However, this mutation has been analyzed *in vitro* and found to reduce binding to serum antibodies and ACE2 [23,24]. Overall, these predictions of additional mutations that may increase the transmissibility of VOCs are supported by previous observations and provide novel predictions of mutations that may emerge as SARS-CoV-2 variants continue to evolve.

## Discussion

As the SARS-CoV-2 pandemic persists and the virus likely becomes endemic, attention must be focused on identifying and managing variants that pose a significant risk to public health. To our knowledge, our models are the first to comprehensively predict the impact of RBD mutations on both ACE2 and human serum antibody affinity. In addition, our models suggest that mutations of the SARS-CoV-2 virus have led to highly transmissible variants that are strongly linked to increased ACE2 affinity and/or human serum antibody escape. This is evidenced by the fact that many RBD mutations identified using our models have already been identified in connection with increased viral transmissibility, particularly L452Q, L452R, T478K, and E484K. Several concerning variants have at least one of these mutations, including Beta, Gamma, Delta, Lambda, Mu, and Omicron.

Additionally, several other mutations that we have identified as concerning have also been observed in other circulating variants. For example, the Lambda variant, which contains the L452Q mutation (along with the T478K mutation), was identified in Peru and has spread widely [25]. Our models predict that the L452Q mutation results in over a two-fold increase in ACE2 affinity and ~2% increase in antibody escape. The Y453F mutation has widely circulated throughout mink populations in the Netherlands, Denmark, and the United States, sparking concern regarding transmission to humans [26, 27]. Our models predict a 3% increase in antibody escape and a modest increase in ACE2 affinity for Y453F. Additionally, the V367F mutant was identified early in 2020 and continues to circulate throughout Europe while the V367A mutation was identified via asymptomatic sampling in the Anhui province in China [3, 20]. Both mutations (V367F and V367A) are predicted by our analysis to increase ACE2 affinity (>twofold increase for V367F), and V367A is also predicted to increase antibody escape by almost 3%.

Beyond accurately identifying mutations with increased transmissibility that have been observed naturally, our models also predict novel mutations that increase transmissibility and which, to the best of our knowledge, have not yet been observed naturally. Future investigation is warranted to experimentally evaluate the impact of these mutations–including our predicted single mutations to VOCs that increase transmissibility–on ACE2 affinity and antibody escape. In addition to experimental validation, future work should be directed towards extending and improving our first-generation models, particularly as it relates to immunity gained through vaccination. As differences have been observed comparing antibody responses elicited from natural infection versus vaccination [28], additional models will be needed in the future to account for such differences. Similarly, as different SARS-CoV-2 vaccines (*e*.*g*., Pfizer-BioNTech, AstraZeneca, Moderna, etc.) have shown different efficacies against SARS-CoV-2 variants, emerging experimental datasets for specific vaccines could also be incorporated into these models. In addition, future work could be extended toward understanding the influence of non-RBD mutations, deleted residues, or co-evolution of mutations, which are not addressed in our models.

Moreover, our machine learning models could be applied toward developing next-generation vaccines and therapeutic antibodies as a valuable complement to experimental efforts [29, 30]. As variant-specific vaccines are already being developed, predictions of RBD mutations that result in large increases in antibody escape, without significantly reducing ACE2 affinity, may be particularly useful toward informing these next-generation vaccine designs [29]. Our models identify several mutations and combinations (e.g., Y453F and E484K) that result in improved antibody escape without a significant tradeoff in ACE2 affinity. Furthermore, recent impressive studies have employed RBD libraries displayed on yeast to map RBD mutations that escape binding to i) clinical monoclonal antibodies (LY-CoV555) and antibody cocktails (Ly-CoV555 and LyCoV016) [31], and ii) monoclonal antibodies specific for distinct RBD epitopes [32]. These training data should further increase the predictive power of machine learning models, which is key for understanding the breadth of neutralizing activity for monoclonal antibodies, antibody cocktails, and vaccine candidates.

It is also important to consider that linear models, like the ones used in this work, cannot capture all context-dependent, non-linear impacts of mutations on various types of protein properties [33, 34]. Therefore, we caution against overinterpreting our findings given that the context-dependent impacts of multisite RBD mutations on ACE2 affinity and antibody escape will, at least in some cases, be incorrectly predicted by our models. Nevertheless, it is notable that our linear models can predict the impacts of two to four RBD mutations on ACE2 affinity and antibody escape with relatively high accuracy despite that all such mutants were removed during the training process (S3 Fig). Our findings are consistent with a recent report that the same type of linear (Ridge Regression) model is adept at reproducing complex behaviors for large mutagenesis datasets, even outperforming complex nonlinear models [35]. It is also important to consider that most of the RBD mutational training data corresponds to reductions in RBD binding to ACE2 and human antibodies, which may be simpler to predict and less context dependent than for RBD mutations that increase binding. Nevertheless, it will be important in the future to experimentally evaluate our predictions of novel multisite RBD mutations, such as the additional RBD mutations in the VOCs, to determine their accuracies.

Overall, machine learning has tremendous potential to complement experimental approaches to improve vaccine and therapeutic development against COVID-19. Our machine learning methods–which predict the impact of RBD mutations on both ACE2 and human serum antibody escape–illustrate this potential via the identification of both novel and circulating SARS-CoV-2 RBD mutations with enhanced transmissibility and should provide a valuable framework for future predictions aimed at mitigating COVID-19.

## Materials and methods

### Data preprocessing

The dataset for ACE2 affinity was preprocessed for preliminary evaluation by averaging experimental measurements of identical sequences [2]. The data was then trimmed to exclude any *K*_*A*,*app*_ measurements below 10^6^ M and above 10^13^ M, which resulted in a final dataset of 64,617 RBD mutants. The dataset for human serum antibody escape, comprised of two to three serum samples from 11 convalescent patients more than 30 days after the onset of symptoms, was preprocessed by averaging repeat values for each RBD mutant for a total of 102,723 RBD mutants [6]. Initial model testing showed such average values increased model accuracy, likely due to outlier smoothing.

### Featurization and model development

All machine learning models were implemented with scikit learn (1.0.2) packages using python (3.8.5). During preliminary investigation, several types of regression models were evaluated, including Ridge Regression, Ordinary least squares, Lasso, Bayesian Ridge, Stochastic Gradient Descent (SGD), Gaussian Process Regression (GPR), Kernel Ridge Radial Basis Function (Kernel Ridge RBF), Kernel Ridge Linear and Elastic Net. Three types of sequence featurization were used, namely one-hot encoding, biophysical descriptors, and amino acid indices, as summarized in S1 Table. The performance evaluation of the initial models is reported in S2 Table, as judged by the average Spearman’s ρ values for fivefold cross-validation. Values used for the three types of featurization are reported in S2 Table. The biophysical descriptors and amino acid indices were chosen to represent a wide range of amino acid properties without substantial overlap. Features within these two sets did not correlate strongly with one another to avoid feature collinearity. For one-hot encoding, RBD sequences were encoded first into a two-dimensional matrix, which was flattened lengthwise into a single dimensional feature vector. One-hot encoding was performed using scikit learn LabelEncoder to numerically encode the amino acid sequences, which could then be translated into binary feature vectors.

Preliminary investigation of models was performed using default parameters for all model architectures, including regularization coefficients, loss terms, and penalties. Both RBF and Linear kernels were tested for Kernel Ridge regression and are reported as separate values (S2 Table). Gaussian Process Regressor was not evaluated for the antibody escape dataset due to extensive computational resources required for this model. The final reported models were linear Ridge Regression models trained with one-hot encoded features (Fig 2). These linear Ridge Regression models were chosen because they had the highest Spearman’s ρ values for the test datasets for both the ACE2 affinity and antibody escape predictions.

Model performance for the final Ridge Regression models for ACE2 affinity and antibody escape was next optimized in two ways, namely further data preprocessing and hyperparameter tuning of the regularization coefficients. For data preprocessing, the sequence count cutoff (minimal number of times the sequence was detected during deep sequencing) was optimized (S5 Fig). Tenfold cross-validation was performed on models with varying sequence count cutoffs, and the highest Spearman’s ρ values of the test sets were chosen as optimal. For the ACE2 affinity model, the optimal sequence cutoff was found to be 53. For the antibody escape model, a sequence count cutoff of 15 was found to be optimal. For hyperparameter tuning, the regularization coefficient (alpha) was optimized. Values across three orders of magnitude were sampled sparsely, with final tuning performed comprehensively for alpha values between 1–10 for the ACE2 affinity model and 1–20 for the antibody escape model. Optimized regularization coefficients of 1.90 for the ACE2 affinity model and 6.2 for the antibody escape model were chosen to maximize the Spearman’s ρ for the test sets. Other hyperparameters were set to default values.

### Virtual scans

To perform the virtual scan of double mutants, a comprehensive set of sequences with two mutations was generated. Only amino acids that were observed at a given site in at least six sequences in the training dataset of both models were sampled in this virtual scan to ensure the models were based on accurate feature weights. This cutoff was chosen by maximizing the predictive capacity of the models for single mutations that were experimentally observed. All single-mutation sequences were withheld from an instance of model training. The Spearman’s ρ value was evaluated for the trained model’s predictions on this withheld test set, varying the sequence observation cutoff from 1 to 25 (S6 Fig). An observation cutoff of six was found to maximize performance of the antibody escape model without significant detrimental effects to the ACE2 affinity model. Therefore, mutations observed experimentally in more than six sequences in both models were included in the comprehensive scan of virtual sequences, sampling all combinations of these single mutations.

The final models were then used to predict ACE2 affinity and antibody escape for these virtual sequences. Sequences reported in the region of concern (S1 Dataset) were identified by limiting predicted ACE2 affinities to values (K_*A*, *app*_) above 4.3x10^10^ M and antibody escape values above 0%. Variants of high concern (S2 Dataset) were identified by further limiting the sequences reported in the ‘Region of high concern’ to those with mutations that could be accomplished via single-nucleotide exchanges without disrupting glycosylation sites and according to equations which may be found in the provided code.

The single-mutation virtual scans of all VOCs were performed by sampling all single mutations (observed in more than six sequences in the training datasets of both algorithms). Predictions of mutations with increases in or maintenance of both properties, which could be accomplished via single-nucleotide exchanges without disrupting glycosylation sites, are reported in S3 Dataset.

### Structural modeling

The crystal structure of the RBD is from the PBD (6MOJ) [16]. PyMOL was used for all structural visualizations.

## Supporting information

S1 Table Summary of the preliminary evaluation of model performance for predicting ACE2 affinity and % human antibody escape using different types of models and featurization methods. Three types of featurization methods were used, namely one-hot encoding (OHE), biophysical descriptors, and amino acid indices. The details of the feature types and values are summarized in S2 Table. Nine types of models were tested, namely ordinary least squares regression, Lasso, Bayesian Ridge, stochastic gradient descent (SGD), gaussian process classifier (GPC), kernel Ridge (radial basis function (RBF) and linear kernels), and elastic net. The average training and test Spearman’s ρ values for each model trained with each type of feature set are reported based on 5-fold cross-validation. Based on this analysis, the Ridge Regression models trained with one-hot encoded features were further optimized and exclusively used in the remainder of this manuscript.
(PDF)

S2 Table Summary of featurization vectors. Feature vectors used in work include (A) one-hot-encoded features, (B) biophysical descriptors, and (C) amino acid index features. Biophysical descriptors and amino acid index values were chosen to represent a wide variety of physicochemical properties while exhibiting low degrees of correlation. RBD sequences were transformed into a matrix of the feature vectors representing the amino acid present at each RBD site. The matrices were then flattened for a final feature set of 1x4020 numerical values.
(PDF)

S1 Fig Ridge Regression model predictions of ACE2 affinity for single RBD mutants are correlated with conventional ACE2 affinity measurements. Predicted ACE2 affinity values (Ridge Regression model with one-hot encoded features) are correlated with conventional measurements of ACE2 affinity for single RBD mutants reported previously [2].
(PDF)

S2 Fig Evaluation of model performance for different biological replicates (ACE2 affinity) and human samples (antibody escape). (A-B) Model displays similar performance (Spearman’s ρ values) in predicting ACE2 affinities for RBD mutants from two biological (experimental) replicates. (C-E) Model predictions of % antibody escape for antibody samples obtained from different convalescent patients. In (C), the worst model performance for predicting % antibody escape from one of the 11 human samples (subject E) is reported. In (D), the best model performance for predicting % antibody escape from one of the 11 human samples (subject J) is reported. In (E), the range of model performances (Spearman’s ρ values) for predicting % antibody escape is reported for the 11 human samples.
(PDF)

S3 Fig Models accurately predict impacts of RBD mutations on ACE2 affinity and % antibody escape for combinations of RBD mutations not observed in the training sets. In each case, the training sets of RBD mutations were filtered to remove all (A, B) double, (C, D) triple, and (E, F) quadruple RBD mutations, and then the models were trained on all remaining RBD mutants that did not contain the combinations of RBD mutations used for training. Finally, the models were tested only on the (A, B) double, (C, D) triple and (E, F) quadruple RBD mutations that were held out of the training process. The goal of this analysis was to evaluate the ability of the models to predict the impacts of combinations of RBD mutations not observed together in the training sets. In each panel, the Spearman’s ρ values are given for the training and test sets.
(PDF)

S4 Fig Evaluation of single RBD mutations with the largest changes in predicted ACE2 affinity and antibody escape achieved via mutation at each RBD residue. (A-B) The largest predicted impact of single mutations on (A) ACE2 affinity and (B) % antibody escape, irrespective of the other property, were evaluated for each RBD residue. Mutation V367F was predicted to have the largest increase in ACE2 affinity, while F456A was found to have the largest increase in antibody escape. V367F has previously been identified in circulation as early as March 2020. F456A has not been widely observed, likely due to the predicted reduction in ACE2 affinity.
(PDF)

S5 Fig Optimization of sequence count cutoffs and hyperparameter tuning. (A-B) Sequence observation cutoffs were optimized for model development to ensure training on accurate data. Optimal cutoffs of (A) 53 counts per RBD mutant for the ACE2 affinity model and (B) 15 counts per RBD mutant for the antibody escape model were identified to maximize the Spearman’s ρ value of the test set predictions with experimental data. (C-D) Hyperparameter tuning of the regularization coefficients revealed optimal values of (C) 1.90 for the ACE2 affinity model and (D) 6.02 for the antibody escape model, which were also identified by maximizing the Spearman’s ρ values for the test sets.
(PDF)

S6 Fig Optimization of RBD mutant sampling during the virtual scan analysis. (A-B) RBD mutation observation cutoffs were optimized for model performance to ensure virtual scans were performed only on sequences with mutations that had been sufficiently observed in the training dataset. (A) No benefit of increasing mutation observations was observed for the ACE2 affinity model. (B) An optimal value of six observations in the training dataset was identified for the antibody escape model in accordance with the maximum Spearman’s ρ values for the test set. All subsequent RBD virtual mutational scans were conducted only for mutations observed in more than six RBD sequences in the training dataset.
(PDF)

S1 Dataset Summary of approximately 5x10^5^ sequences for RBD double mutants that fall within the region of concern (S4 Fig). (CSV)

S2 Dataset Summary of twenty-nine double mutants with the highest predicted increases in ACE2 affinity and antibody escape, located as far from wild-type behavior as possible, that were considered ‘variants of high concern’. (XLSX)

S3 Dataset Summary of single RBD mutations to VOCs that can be achieved with single nucleotide exchanges without disrupting glycosylation sites and which increase either ACE2 affinity or antibody escape while maintaining or increasing the other property (Fig 4A). (XLSX)
