# Supplementary material for: Mutational analysis of SARS-CoV-2 variants of concern reveals key tradeoffs between receptor affinity and antibody escape
Source: PLoS Comput Biol. 2022 May 31;18(5):e1010160. doi: 10.1371/journal.pcbi.1010160 (PMC9223403; doi:10.1371/journal.pcbi.1010160)
Supplement: S1 Table — Three types of featurization methods were used, namely one-hot encoding (OHE), biophysical descriptors, and amino acid indices. The details of the feature types and values are summarized in S2 Table. Nine types of models were tested, namely ordinary least squares regression, Lasso, Bayesian Ridge, stochastic gradient descent (SGD), gaussian process classifier (GPC), kernel Ridge (radial basis function (RBF) and linear kernels), and elastic net. The average training and test Spearman’s ρ values for each model trained with each type of feature set are reported based on 5-fold cross-validation. Based on this analysis, the Ridge Regression models trained with one-hot encoded features were further optimized and exclusively used in the remainder of this manuscript. (PDF) [file pcbi.1010160.s001.pdf]

Table S1. Summary of the preliminary evaluation of model performance for predicting ACE2 affinity and % human antibody escape using different types of models and featurization methods. Three types of featurization methods were used, namely one-hot encoding (OHE), biophysical descriptors, and amino acid indices. The details of the feature types and values are summarized in Table S2. Nine types of models were tested, namely ordinary least squares regression, Lasso, Bayesian Ridge, stochastic gradient descent (SGD), gaussian process classifier (GPC), kernel Ridge (radial basis function (RBF) and linear kernels), and elastic net. The average training and test Spearman's ρ values for each model trained with each type of feature set are reported based on 5-fold cross-validation. Based on this analysis, the Ridge Regression models trained with one-hot encoded features were further optimized and exclusively used in the remainder of this manuscript.

**ACE2 affinity models**			**Human serum antibody escape models**		
**One hot encoding**	Training data Spearman's ρ	Test data Spearman's ρ	**One hot encoding**	Training data Spearman's ρ	Test data Spearman's ρ
Ridge Regression	0.94	0.93	Ridge Regression	0.69	0.66
Ordinary least squares	0.93	0.81	Ordinary least squares	0.67	0.45
Lasso	0.94	0.92	Lasso	0.68	0.66
Bayesian Ridge	0.94	0.93	Bayesian Ridge	0.69	0.66
SGD	0.84	0.81	SGD	0.58	0.56
GPR	0.99	0.57	GPR	0.99	0.04
Kernel Ridge (RBF)	0.62	0.6	Kernel Ridge (RBF)	0.47	0.45
Kernel Ridge (Linear)	0.93	0.85	Kernel Ridge (Linear)	0.69	0.5
Elastic Net	0.93	0.89	Elastic Net	0.64	0.57
**Biophysical descriptors**	Training data Spearman's ρ	Test data Spearman's ρ	**Biophysical descriptors**	Training data Spearman's ρ	Test data Spearman's ρ
Ridge Regression	0.85	0.84	Ridge Regression	0.64	0.62
Ordinary least squares	0.84	0.79	Ordinary least squares	0.64	0.62
Lasso	0.94	0.93	Lasso	0.63	0.62
Bayesian Ridge	0.84	0.79	Bayesian Ridge	0.63	0.62
SGD	0	0	SGD	0	0.1
GPR	0.99	0.34	GPR	0.99	-0.24
Kernel Ridge (RBF)	0.77	0.74	Kernel Ridge (RBF)	0.58	0.52
Kernel Ridge (Linear)	0.77	0.74	Kernel Ridge (Linear)	0.61	0.52
Elastic Net	0.84	0.8	Elastic Net	0.61	0.53
**Amino acid indices**	Training data Spearman's ρ	Test data Spearman's ρ	**Amino acid indices**	Training data Spearman's ρ	Test data Spearman's ρ
Ridge Regression	0.88	0.87	Ridge Regression	0.65	0.63
Ordinary least squares	0.87	0.81	Ordinary least squares	0.65	0.63
Lasso	0.88	0.87	Lasso	0.64	0.63
Bayesian Ridge	0.87	0.86	Bayesian Ridge	0.65	0.63
SGD	0.04	0.04	SGD	-0.01	0
GPR	0.99	0.33	GPR	N/A	N/A
Kernel Ridge (RBF)	0.65	0.48	Kernel Ridge (RBF)	0.8	0.01
Kernel Ridge (Linear)	0.86	0.82	Kernel Ridge (Linear)	0.62	0.56
Elastic Net	0.87	0.82	Elastic Net	0.65	0.63
**Combined feature sets**	Training data Spearman's ρ	Test data Spearman's ρ	**Combined feature sets**	Training data Spearman's ρ	Test data Spearman's ρ
Ridge Regression	0.94	0.92	Ridge Regression	0.69	0.64
