# Supplementary material for: Mutational analysis of SARS-CoV-2 variants of concern reveals key tradeoffs between receptor affinity and antibody escape
Source: PLoS Comput Biol. 2022 May 31;18(5):e1010160. doi: 10.1371/journal.pcbi.1010160 (PMC9223403; doi:10.1371/journal.pcbi.1010160)
Supplement: S2 Table — Feature vectors used in work include (A) one-hot-encoded features, (B) biophysical descriptors, and (C) amino acid index features. Biophysical descriptors and amino acid index values were chosen to represent a wide variety of physicochemical properties while exhibiting low degrees of correlation. RBD sequences were transformed into a matrix of the feature vectors representing the amino acid present at each RBD site. The matrices were then flattened for a final feature set of 1x4020 numerical values. (PDF) [file pcbi.1010160.s002.pdf]

Table S2. Summary of featurization vectors. Feature vectors used in work include (A) one-hot-encoded features, (B) biophysical descriptors, and (C) amino acid index features. Biophysical descriptors and amino acid index values were chosen to represent a wide variety of physicochemical properties while exhibiting low degrees of correlation. RBD sequences were transformed into a matrix of the feature vectors representing the amino acid present at each RBD site. The matrices were then flattened for a final feature set of 1x4020 numerical values.

A

Residue	Feature Vector																			
A	1	0	0	0	0	0	0	0	0	0	0	0	0	0	0	0	0	0	0	0
C	0	1	0	0	0	0	0	0	0	0	0	0	0	0	0	0	0	0	0	0
D	0	0	1	0	0	0	0	0	0	0	0	0	0	0	0	0	0	0	0	0
E	0	0	0	1	0	0	0	0	0	0	0	0	0	0	0	0	0	0	0	0
F	0	0	0	0	1	0	0	0	0	0	0	0	0	0	0	0	0	0	0	0
G	0	0	0	0	0	1	0	0	0	0	0	0	0	0	0	0	0	0	0	0
H	0	0	0	0	0	0	1	0	0	0	0	0	0	0	0	0	0	0	0	0
I	0	0	0	0	0	0	0	1	0	0	0	0	0	0	0	0	0	0	0	0
K	0	0	0	0	0	0	0	0	1	0	0	0	0	0	0	0	0	0	0	0
L	0	0	0	0	0	0	0	0	0	1	0	0	0	0	0	0	0	0	0	0
M	0	0	0	0	0	0	0	0	0	0	1	0	0	0	0	0	0	0	0	0
N	0	0	0	0	0	0	0	0	0	0	0	1	0	0	0	0	0	0	0	0
P	0	0	0	0	0	0	0	0	0	0	0	0	1	0	0	0	0	0	0	0
Q	0	0	0	0	0	0	0	0	0	0	0	0	0	1	0	0	0	0	0	0
R	0	0	0	0	0	0	0	0	0	0	0	0	0	0	1	0	0	0	0	0
S	0	0	0	0	0	0	0	0	0	0	0	0	0	0	0	1	0	0	0	0
T	0	0	0	0	0	0	0	0	0	0	0	0	0	0	0	0	1	0	0	0
V	0	0	0	0	0	0	0	0	0	0	0	0	0	0	0	0	0	1	0	0
W	0	0	0	0	0	0	0	0	0	0	0	0	0	0	0	0	0	0	1	0
Y	0	0	0	0	0	0	0	0	0	0	0	0	0	0	0	0	0	0	0	1

B

Residue	Positive charge	Negative charge	Hydropathy score	pI	# Side chain atoms	Hydrogen bond D/A
A	0	0	1.8	6.0	1	0
C	0	0	2.5	5.1	2	0
D	0	1	-3.5	2.8	4	1
E	0	1	-3.5	3.2	5	1
F	0	0	2.8	5.5	7	0
G	0	0	-0.4	6.0	0	0
H	1	0	-3.2	7.6	6	1
I	0	0	4.5	6.0	4	0
K	1	0	-3.9	9.7	5	1
L	0	0	3.8	6.0	4	0
M	0	0	1.9	5.7	4	0
N	0	0	-3.5	5.4	4	0
P	0	0	-1.6	6.5	3	0
Q	0	0	-3.5	5.7	5	0
R	1	0	-4.5	10.8	7	0
S	0	0	-0.8	5.7	2	1
T	0	0	-0.7	5.9	3	1
V	0	0	4.2	6.0	3	0
W	0	0	-0.9	6.0	10	0
Y	0	0	-1.3	5.7	8	1

C

Residue	BHAR880101 Average flexibility indices	BIGC670101 residue volume	CHOC760102 Residue accessible surface area in folded protein	EISD860101 Solvation free energy	CHAM830107 charge transfer capability	CHAM830108 A parameter of charge transfer donor capability	KANM800101 Average relative probability of helix	KANM800102 Average relative probability of beta-sheet
A	0.357	52.6	25.0	0.7	0.0	0.0	1.36	0.81
C	0.365	102	23.0	1.9	0.0	0.0	1.21	1.24
D	0.529	109.1	90.0	-2.1	0.0	1.0	1	0.85
E	0.466	105.1	97.0	-0.6	0.0	1.0	1.22	0.77
F	0.463	75.7	63.0	-0.6	1.0	1.0	0.89	0.62
G	0.295	97.7	31.0	2.4	0.0	1.0	1.45	1.05
H	0.511	68.4	50.0	-1.2	1.0	0.0	1.04	0.71
I	0.314	113.9	24.0	2.3	0.0	1.0	1.05	1.2
K	0.346	68.3	19.0	0.4	0.0	1.0	0.82	1.17
L	0.509	73.6	50.0	1.2	0.0	0.0	0.52	0.61
M	0.493	89.7	71.0	-0.2	0.0	1.0	1.14	0.98
N	0.507	54.9	44.0	0.0	0.0	0.0	0.74	0.92
P	0.497	84.7	49.0	-0.8	1.0	0.0	1.48	0.53
Q	0.444	71.2	47.0	0.5	0.0	0.0	0.81	1.18
R	0.544	36.3	23.0	0.0	1.0	0.0	0.63	0.88
S	0.305	135.4	32.0	2.6	0.0	1.0	0.97	1.18
T	0.323	91.9	43.0	0.6	0.0	1.0	1.11	0.92
V	0.42	116.2	60.0	1.6	0.0	1.0	0.79	1.23
W	0.462	102	18.0	1.9	0.0	0.0	1.08	1.48
Y	0.386	85.1	18.0	1.5	0.0	0.0	0.94	1.66
