# Supplementary figures and images for: Mutational analysis of SARS-CoV-2 variants of concern reveals key tradeoffs between receptor affinity and antibody escape
Source: PLoS Comput Biol. 2022 May 31;18(5):e1010160. doi: 10.1371/journal.pcbi.1010160 (PMC9223403; doi:10.1371/journal.pcbi.1010160)

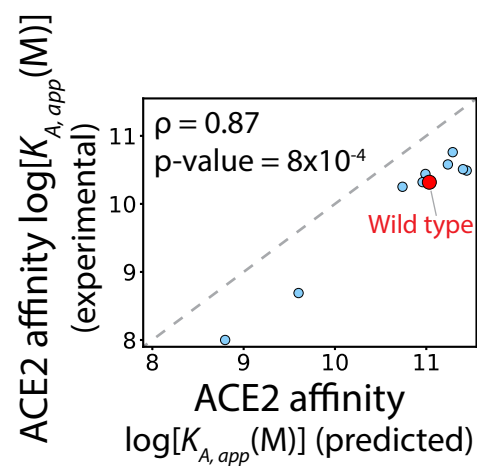

Supplement: S1 Fig — Predicted ACE2 affinity values (Ridge Regression model with one-hot encoded features) are correlated with conventional measurements of ACE2 affinity for single RBD mutants reported previously [2]. (PDF) [file pcbi.1010160.s003.pdf]
