# Supplementary material for: Mutational analysis of SARS-CoV-2 variants of concern reveals key tradeoffs between receptor affinity and antibody escape
Source: PLoS Comput Biol. 2022 May 31;18(5):e1010160. doi: 10.1371/journal.pcbi.1010160 (PMC9223403; doi:10.1371/journal.pcbi.1010160)
Supplement: S2 Fig — (A-B) Model displays similar performance (Spearman’s ρ values) in predicting ACE2 affinities for RBD mutants from two biological (experimental) replicates. (C-E) Model predictions of % antibody escape for antibody samples obtained from different convalescent patients. In (C), the worst model performance for predicting % antibody escape from one of the 11 human samples (subject E) is reported. In (D), the best model performance for predicting % antibody escape from one of the 11 human samples (subject J) is reported. In (E), the range of model performances (Spearman’s ρ values) for predicting % antibody escape is reported for the 11 human samples. (PDF) [file pcbi.1010160.s004.pdf]

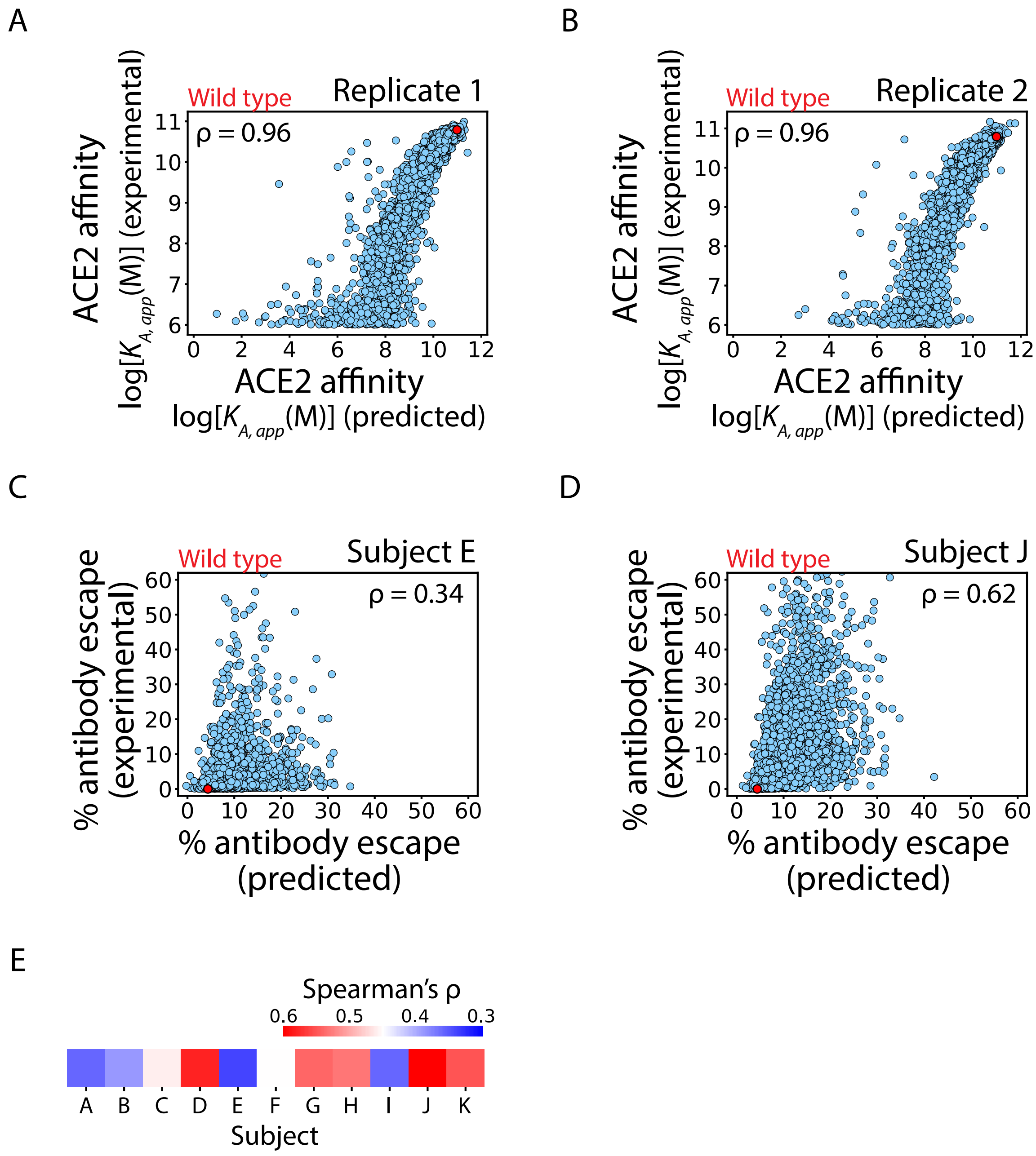

Figure S2. Evaluation of model performance for different biological replicates (ACE2 affinity) and human samples (antibody escape). (A-B) Model displays similar performance (Spearman's ρ values) in predicting ACE2 affinities for RBD mutants from two biological (experimental) replicates. (C-E) Model predicts % antibody escape for antibody samples obtained from different convalescent patients with different Spearman's ρ values. In (C), the worst model performance for predicting % antibody escape from one of the 11 human samples (subject E) is reported. In (D), the best model performance for predicting % antibody escape from one of the 11 human samples (subject J) is reported. In (E), the range of model performances (Spearman's ρ values) for predicting % antibody escape is reported for the 11 human samples.
