# Supplementary material for: Mutational analysis of SARS-CoV-2 variants of concern reveals key tradeoffs between receptor affinity and antibody escape
Source: PLoS Comput Biol. 2022 May 31;18(5):e1010160. doi: 10.1371/journal.pcbi.1010160 (PMC9223403; doi:10.1371/journal.pcbi.1010160)
Supplement: S3 Fig — In each case, the training sets of RBD mutations were filtered to remove all (A, B) double, (C, D) triple, and (E, F) quadruple RBD mutations, and then the models were trained on all remaining RBD mutants that did not contain the combinations of RBD mutations used for training. Finally, the models were tested only on the (A, B) double, (C, D) triple and (E, F) quadruple RBD mutations that were held out of the training process. The goal of this analysis was to evaluate the ability of the models to predict the impacts of combinations of RBD mutations not observed together in the training sets. In each panel, the Spearman’s ρ values are given for the training and test sets. (PDF) [file pcbi.1010160.s005.pdf]

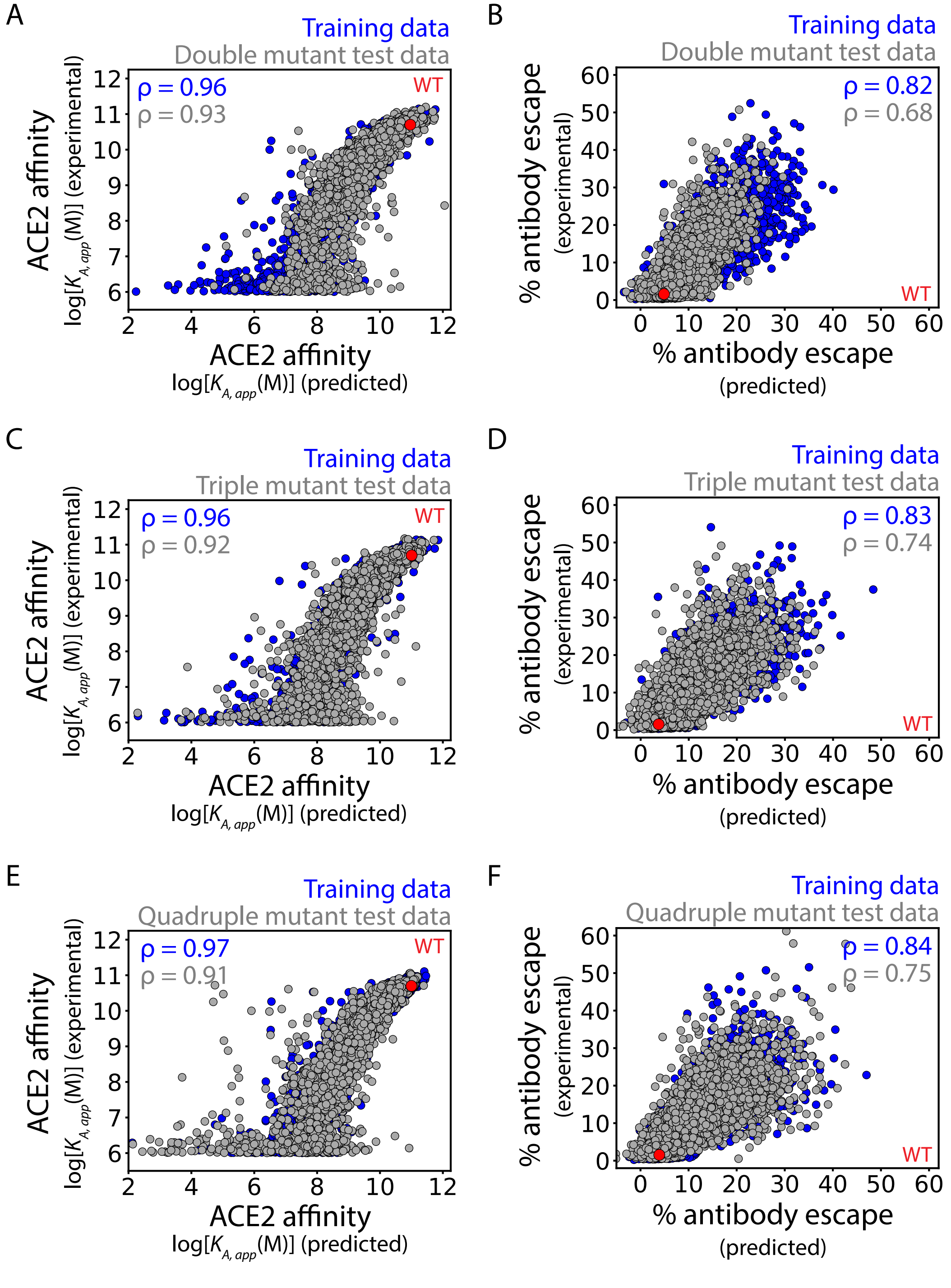

Figure S3. Models accurately predict impacts of RBD mutations on ACE2 affinity and % antibody escape for combinations of RBD mutations not observed in the training sets. In each case, the training sets of RBD mutations were filtered to remove all (A, B) double, (C, D) triple, and (E, F) quadruple RBD mutations, and then the models were trained on all remaining RBD mutants that did not contain the combinations of RBD mutations used for training. Finally, the models were tested only on the (A, B) double, (C, D) triple and (E, F) quadruple RBD mutations that were held out of the training process. The goal of this analysis was to evaluate the ability of the models to predict the impacts of combinations of RBD mutations never observed together in the training sets. In each panel, the Spearman's ρ values are given for the training and test sets.
