# Supplementary material for: Mutational analysis of SARS-CoV-2 variants of concern reveals key tradeoffs between receptor affinity and antibody escape
Source: PLoS Comput Biol. 2022 May 31;18(5):e1010160. doi: 10.1371/journal.pcbi.1010160 (PMC9223403; doi:10.1371/journal.pcbi.1010160)
Supplement: S4 Fig — (A-B) The largest predicted impact of single mutations on (A) ACE2 affinity and (B) % antibody escape, irrespective of the other property, were evaluated for each RBD residue. Mutation V367F was predicted to have the largest increase in ACE2 affinity, while F456A was found to have the largest increase in antibody escape. V367F has previously been identified in circulation as early as March 2020. F456A has not been widely observed, likely due to the predicted reduction in ACE2 affinity. (PDF) [file pcbi.1010160.s006.pdf]

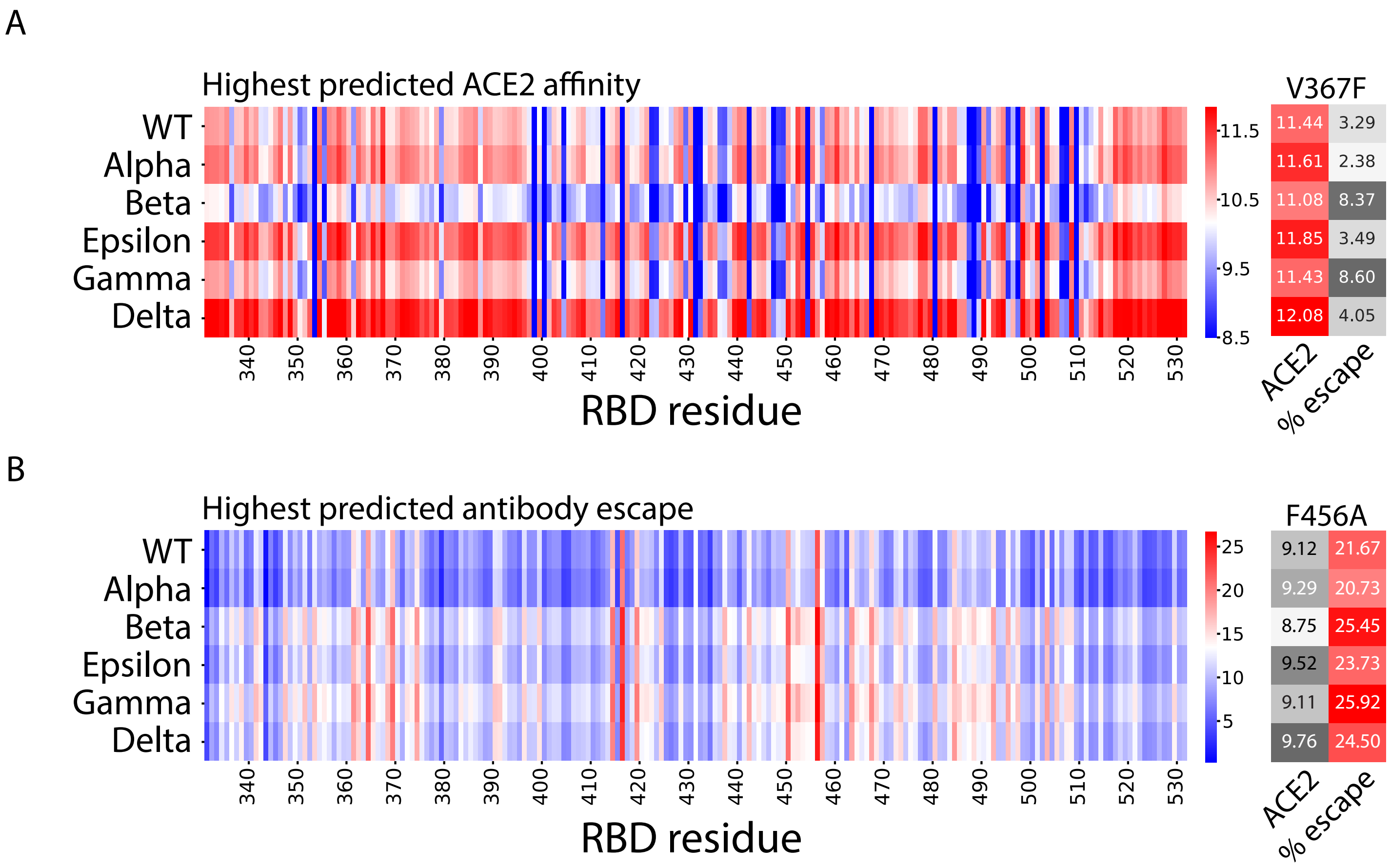

Figure S4. Evaluation of single RBD mutations with the largest changes in predicted ACE2 affinity and human antibody escape achieved via mutation at each RBD residue. (A-B) The largest predicted impact of single mutations on (A) ACE2 affinity and (B) % antibody escape, irrespective of the other property, were evaluated for each RBD residue. Mutation V367F was predicted to have the largest increase in ACE2 affinity, while F456A was found to have the largest increase in antibody escape. V367F has previously been identified in circulation as early as March 2020. F456A has not been widely observed, likely due to the predicted reduction in ACE2 affinity.
