# Supplementary material for: Mutational analysis of SARS-CoV-2 variants of concern reveals key tradeoffs between receptor affinity and antibody escape
Source: PLoS Comput Biol. 2022 May 31;18(5):e1010160. doi: 10.1371/journal.pcbi.1010160 (PMC9223403; doi:10.1371/journal.pcbi.1010160)
Supplement: S5 Fig — (A-B) Sequence observation cutoffs were optimized for model development to ensure training on accurate data. Optimal cutoffs of (A) 53 counts per RBD mutant for the ACE2 affinity model and (B) 15 counts per RBD mutant for the antibody escape model were identified to maximize the Spearman’s ρ value of the test set predictions with experimental data. (C-D) Hyperparameter tuning of the regularization coefficients revealed optimal values of (C) 1.90 for the ACE2 affinity model and (D) 6.02 for the antibody escape model, which were also identified by maximizing the Spearman’s ρ values for the test sets. (PDF) [file pcbi.1010160.s007.pdf]

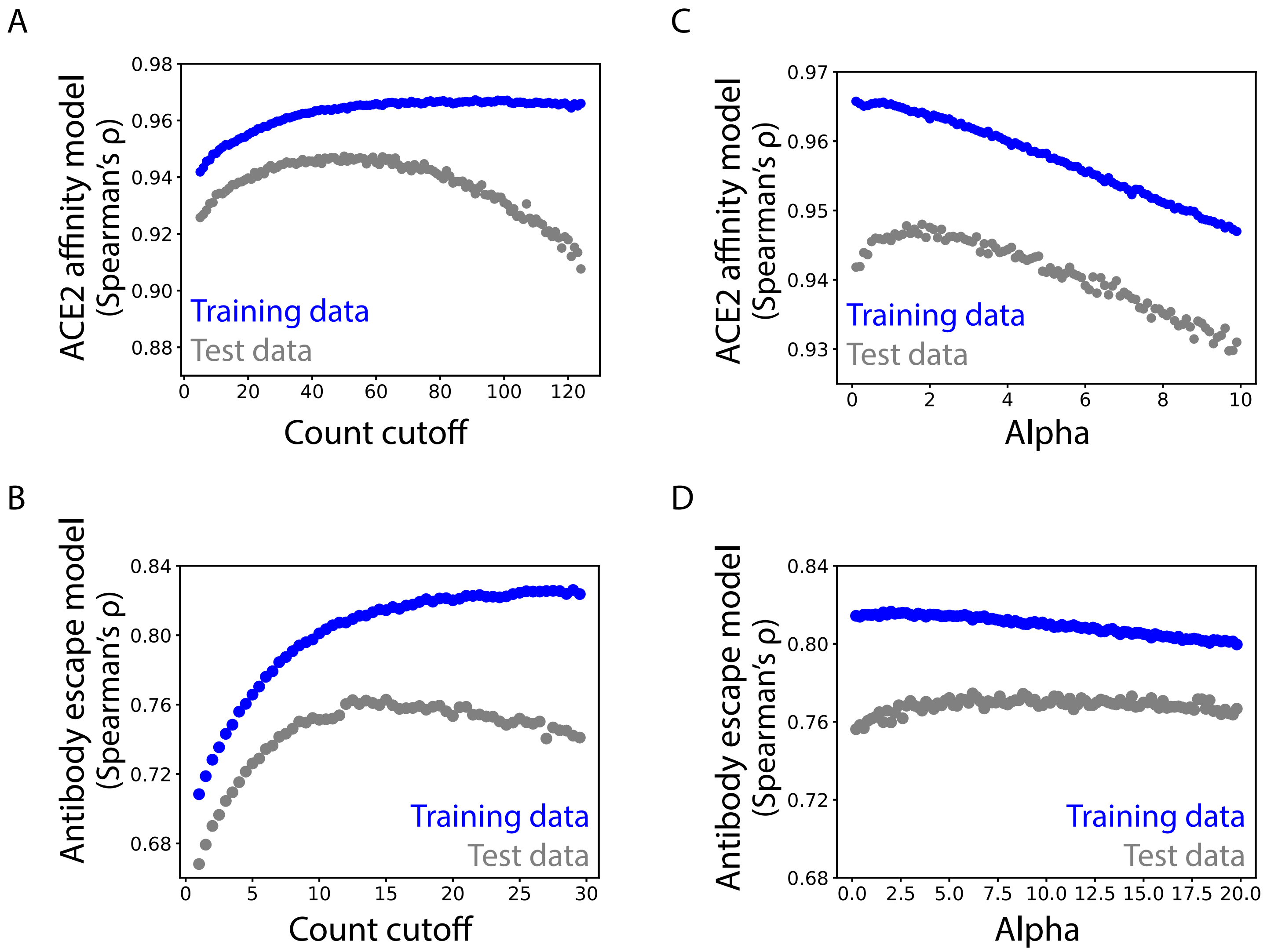

Figure S5. Optimization of sequence count cutoffs and hyperparameter tuning. (A-B) Sequence observation cutoffs were optimized for model development to ensure training on accurate data. Optimal cutoffs of (A) 53 counts per RBD mutant for the ACE2 affinity model and (B) 15 counts per RBD mutant for the antibody escape model were identified to maximize the Spearman's ρ of the test set predictions with experimental data. (C-D) Hyperparameter tuning of the regularization coefficient revealed optimal values of (C) 1.90 for the ACE2 affinity model and (D) 6.02 for the human antibody escape model, which were also identified by maximizing the test set Spearman's ρ values.
