# Supplementary material for: Mutational analysis of SARS-CoV-2 variants of concern reveals key tradeoffs between receptor affinity and antibody escape
Source: PLoS Comput Biol. 2022 May 31;18(5):e1010160. doi: 10.1371/journal.pcbi.1010160 (PMC9223403; doi:10.1371/journal.pcbi.1010160)
Supplement: S6 Fig — (A-B) RBD mutation observation cutoffs were optimized for model performance to ensure virtual scans were performed only on sequences with mutations that had been sufficiently observed in the training dataset. (A) No benefit of increasing mutation observations was observed for the ACE2 affinity model. (B) An optimal value of six observations in the training dataset was identified for the antibody escape model in accordance with the maximum Spearman’s ρ values for the test set. All subsequent RBD virtual mutational scans were conducted only for mutations observed in more than six RBD sequences in the training dataset. (PDF) [file pcbi.1010160.s008.pdf]

A

B

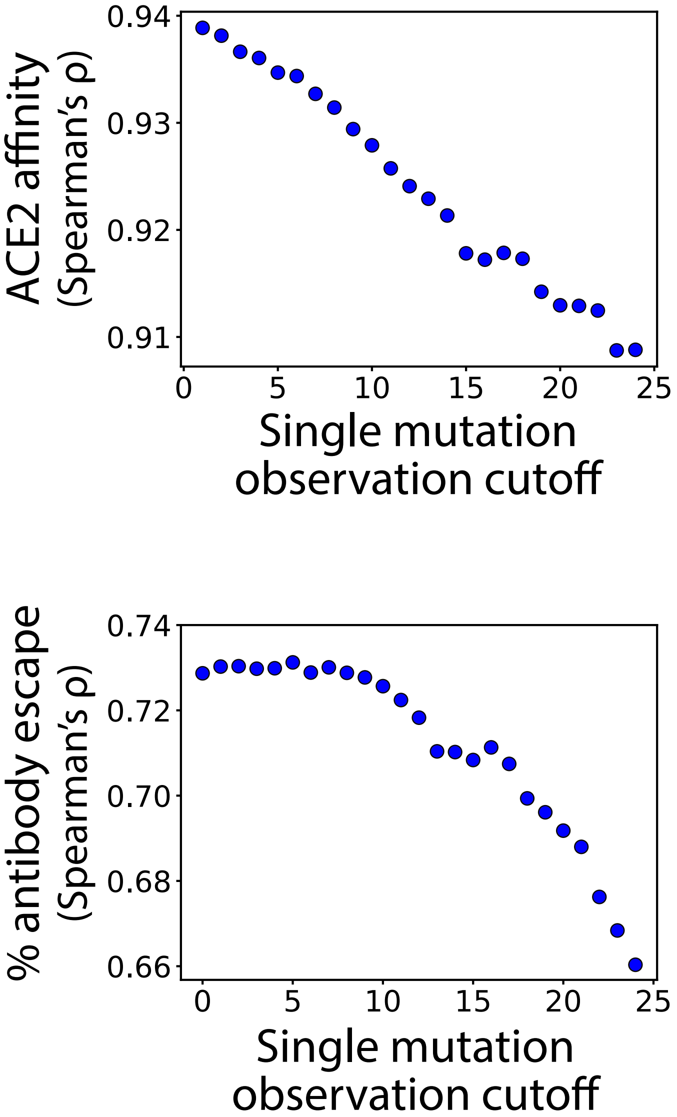


**Figure S6. Optimization of sampling of RBD mutants during the virtual scan analysis.** (A-B) RBD mutation observation cutoffs were optimized for model performance to ensure virtual scans were performed only on sequences with mutations that had been sufficiently observed in the training dataset. (A) No benefit of increasing mutation observations was observed for the ACE2 affinity model. (B) An optimal value of six observations in the training dataset was identified for the antibody escape model in accordance with the maximum Spearman's ρ values for the test set. All subsequent RBD virtual mutational scans were conducted only for mutations observed in more than six RBD sequences in the training dataset.
